# Molecular and Phylogeographic Analysis of Human Immuno-deficiency Virus Type 1 Strains Infecting Treatment-naive Patients from Kigali, Rwanda

**DOI:** 10.1371/journal.pone.0042557

**Published:** 2012-08-14

**Authors:** John Rusine, Suzanne Jurriaans, Janneke van de Wijgert, Marion Cornelissen, Brenda Kateera, Kimberly Boer, Etienne Karita, Odette Mukabayire, Menno de Jong, Pascale Ondoa

**Affiliations:** 1 National Reference Laboratory, Kigali, Rwanda; 2 Laboratory of Virology, Department of Medical Microbiology, Academic Medical Center, University of Amsterdam, Amsterdam Institute for Global Health and Development (AIGHD), Amsterdam, The Netherlands; 3 Department of Global Health, Academic Medical Center, University of Amsterdam, Amsterdam Institute for Global Health and Development (AIGHD), Amsterdam, The Netherlands; 4 The Infectious Diseases Network for Treatment and Research in Africa (INTERACT) Project, Kigali, Rwanda; 5 Royal Tropical Institute (KIT), Biomedical Research, Epidemiology Unit, Amsterdam Institute for Global Health and Development (AIGHD), Amsterdam, The Netherlands; 6 Projet San Francisco, Kigali, Rwanda; National HIV and Retrovirology Laboratories, Canada

## Abstract

This study aimed at describing the genetic subtype distribution of HIV-1 strains circulating in Kigali and their epidemiological link with the HIV-1 strains from the five countries surrounding Rwanda. One hundred and thirty eight *pol* (*RT* and *PR*) sequences from 116 chronically- and 22 recently-infected antiretroviral therapy (ART)-naïve patients from Kigali were generated and subjected to HIV drug resistance (HIV-DR), phylogenetic and recombinant analyses in connection with 366 reference *pol* sequences from Rwanda, Burundi, Kenya, Democratic Republic of Congo, Tanzania and Uganda (Los Alamos database). Among the Rwandan samples, subtype A1 predominated (71.7%), followed by A1/C recombinants (18.1%), subtype C (5.8%), subtype D (2.9%), one A1/D recombinant (0.7%) and one unknown subtype (0.7%). Thirteen unique and three multiple A1/C recombinant forms were identified. No evidence for direct transmission events was found within the Rwandan strains. Molecular characteristics of HIV-1 were similar between chronically and recently-infected individuals and were not significantly associated with demographic or social factors. Our report suggests that the HIV-1 epidemic in Kigali is characterized by the emergence of A1/C recombinants and is not phylogenetically connected with the HIV-1 epidemic in the five neighboring countries. The relatively low level of transmitted HIV-DR mutations (2.9%) reported here indicates the good performance of the ART programme in Rwanda. However, the importance of promoting couples' counseling, testing and disclosure during HIV prevention strategies is highlighted.

## Introduction

Rwanda is one of the African countries most affected by HIV/AIDS [1]. Important fluctuations of the national HIV prevalence have been observed in this country since the first HIV infection case was reported in 1983 [2]. The initial estimation of the national HIV prevalence rate in between 1988 and 1989 was 17.8% among urban and 1.3% among rural populations [3], [4]. In 1996 and immediately after the civil war, the national HIV prevalence reached 27% among the urban and 6.9% among the rural population [5], [6]. This peak has been followed by an overall decline of the national HIV prevalence since the late 1990's, most likely due to an improved serosurveillance strategy and to the important resources devoted to the fight against HIV/AIDS in Rwanda.

Frequent human migration in the region, might have resulted in complex routes of HIV transmission. Population groups have traditionally been moving inside and outside Rwanda for economical, spatial, and political reasons since the early 20^th^ century. In the past decades, war and political turmoil have led to further population shifts in the country with millions of Rwandans fleeing their home to surrounding countries [7], [8] Nowadays, 80% of the Rwandan population returned from the diaspora [9], [10] mainly to urban areas.

Similarly to Rwanda, the five neighboring countries where Rwandan populations have been moving in and out are all characterized by a high HIV prevalence: from 8% in Kenya to 20% in Uganda [11]. The region is also characterized by a very heterogeneous epidemic. Uganda and Kenya show a predominance of subtype A and D [12], [13], [14]. In Burundi, the epidemic is largely due to subtype C [15] while subtype A and C predominate in Tanzania [16], [17]. The Democratic Republic of Congo, the presumed epicenter of the epidemic, shows a highly heterogeneous distribution of eight HIV subtypes, with a predominance of subtypes A, G and D and multiple unique circulating recombinant forms [18], [19]. In Rwanda, information on HIV-1 subtypes distribution is scarce and indicates a large predominance of subtype A (or A1) followed by subtype C and with a discreet presence of subtype D and A/C recombinants [20], [21]. HIV-1 diversity translates into the classification of the virus into groups, subtypes, sub-subtypes and circulating recombinant forms that are generally associated with particular geographic region, risk groups and mode of transmission. Different HIV-1 subtypes are reported to impact on the viral fitness, immunogenicity and pathogenicity, which might have consequences for prophylaxis and therapeutic interventions [22]. Studying the distribution of HIV subtypes within a given population provides the opportunity to study patterns and trends of the epidemic and track routes of HIV transmission [23].

The high mutation rate of HIV can also lead to the emergence of drug resistant strains under the selective pressure of antiretroviral (ARV) drugs. Drug resistant viruses can subsequently be transmitted with serious implications in terms of efficacy of first line antiretroviral therapy (ART). Recent and particularly alarming data from neighboring Uganda [24] suggest that baseline HIV drug resistance (HIV-DR) may increase in Rwanda via population movements across the border. Although the roll out of prevention of mother-to-child transmission (PMTCT) and ART have been initiated in Rwanda in 1999 and 2004 [25], respectively, information on the level of transmitted HIV-DR circulating in the country is still scarce.

Genotypic HIV resistance tests provide a source of HIV *pol* sequence data that are used to screen for HIVDR mutations and determine HIV-1 subtypes. The *pol* sequences can also be used to conduct phylogeographic analyses, a useful tool to examine the epidemiology of HIV in terms of relationships between groups of infected persons.

The heterogeneous HIV-1 subtype distribution in East Africa facilitates the study of HIV epidemiology in the region. To our knowledge, little data on the molecular characteristics of HIV have been generated for Rwanda. In addition, few studies have specifically examined the phylogeographic characteristics of HIV circulating in Rwanda, in relation to the current HIV epidemics of the five neighboring countries. From a public health point of view, the identification of the main entry or focal points of HIV transmission is important to support the implementation of efficient prevention strategies that target relevant populations.

This study was conducted to describe the genetic subtypes of HIV-1 strains derived from infected individuals from Kigali, Rwanda using *protease* (*PR*) and *reverse transcriptase (RT)* genes sequences. To gain more insight into the dynamic of the epidemic in Kigali, our aim was to identify clusters of infections amongst chronically and recently HIV-infected Rwandans. We also analyzed the epidemiological links between HIV strains circulating in Rwanda and in its five neighboring countries. In addition, the *pol* sequence information was used to examine whether HIV-DR mutations are being transmitted within and across the borders of Rwanda.

Demographic and social factors associated with particular HIV genetic characteristics, as well as differences between chronic and recent infections were investigated.

## Materials and Methods

### Ethics statement

Written informed consent was obtained from all patients. Patients aged 18 to 20 also obtained parental/guardian consent for participation. Illiterate persons were requested to provide a thumbprint witnessed by a person independent of the study staff. The studies were approved by the Rwandan National Ethical Committee (RNEC).

### Study population

Chronically HIV-1-infected individuals were recruited from two sources:

1. The voluntary counseling and testing (VCT) program at the outpatient clinic of the Treatment and Research AIDS Center (TRAC/Plus) in Kigali between November 2007 and September 2009. The recruitment took place in the frame of the SEARCH (Side Effect and Reproductive Health in a cohort on HAART) study examining the impact of highly active antiretroviral treatment (HAART) on various aspects of reproductive health in HIV-infected men and women and the incidence of clinically important adverse effects of HAART. HIV testing was done by First Response Rapid Test (Premier Medical Corporation, India) and Uni-Gold Rapid Test (Trinity BiotechPlc, Ireland), with Capillus HIV-1/HIV-2 Rapid Test (TrinityBiotech Plc, Ireland) as the tie-breaker. Rapid test-positive results were confirmed by Murex HIV Ag/Ab Combination ELISA (Abbott Laboratories, Germany). Individuals testing positive for HIV-1, clinically stable and immediately eligible for ART according to the Rwandan national guidelines [26] were asked to participate in the study. Other inclusion criteria included being ART-naïve; residing, and planning to reside within travel distance from the TRAC clinic; willing and able to adhere to study protocol and able to give informed consent for enrolment in the study. For each patient eligible for ART, the chronic character of infection (i.e infection older than 180 days) was retrospectively confirmed by the presence of any of the following: CD4 count <350 cells/mL, WHO stage≥II or first positive HIV serology more than six month ago. The main exclusion criteria were: not immediately eligible for ART, not ART-naïve, any clinical suspicion or any laboratory diagnosis of active tuberculosis, no evidence for chronic HIV-1 infection or being pregnant. Previous use of ARV for PMTCT was not an exclusion criterion. Patients were retrospectively selected for this study based on their completion of the 12 month visit post initiation of therapy and the availability of plasma samples for genotypic analysis.
2. A cross-sectional HIV testing survey amongst women with a high risk for HIV through sexual exposure, i.e individuals having exchanged sex for money at least once in the last month and/or currently having sex with multiple partners plus having sex at least twice per week. Study participants were recruited at Projet Ubuzima, a not-for-profitorganization for medical research, with an onsite research clinic and laboratory in Kigali, Rwanda and are described elsewhere [27], [28]. Samples from participants who tested positive for HIV using the testing algorithm described above, were further investigated using the capture enzyme immunoassay (BED) (CalypteH Biomedical Corporation, Oregon, US) [29] and AxSYM Avidity Index method (Ax–AI) (Abbott, USA) [30], [31]. The BED assay measures the ratio of HIV-specific immunoglobulin (IgG) antibody to total antibody; a low proportion indicates infection within the past 155 days, i.e., recent infection (95% confidence interval (CI): 146–165) The Ax-AI method measures the “avidity” or the strength of the HIV antibody-antigen bond; avidity is weak among individuals infected during the past 180 days (i.e., recent infection). The BED and the Ax-AI assay testing in these patients were performed according to the manufacturers' instructions and have been described elsewhere [24]. Patients that were simultaneously positive with the BED and the Ax-AI assays (i.e, not compatible with recent infection) were considered as chronically infected. Patients that were simultaneously negative with the BED and the Ax-AI assays were considered as recently infected.

Recently HIV-1-infected individuals were recruited from the cross-sectional survey described above and selected in two ways:

1. Women who tested ELISA positive in the cross-sectional survey, that were concordant negative for both the BED and the Ax–AI assay and hence classified as recent infections. Only non pregnant women were selected.
2. Women who tested ELISA negative in the cross sectional HIV testing survey described above. HIV negative women that were not pregnant were eligible to participate in prospective observational HIV incidence cohort described elsewhere [24], [25]. Negative plasma were pooled and tested by HIV RNA PCR (Cobas Amplicor) to exclude HIV acute infection. Study participants returned for quarterly follow-up visits for one year and then for a single visit during the second year of follow up. All women were receiving counseling and they were screened for HIV during their visits, using the algorithm described above. Women that seroconverted during the study were confirmed by PCR test; as well their previous follow-up sample was re-checked to confirm negative status. This certified seroconversion within a three month period, hence recent infection.

### Demographic data

Age, gender, marital status, information on the sexual behavior and previous use of PMTCT were collected from all study participants at study entry. A clinical examination to screen for the presence of HIV symptoms was also done.

### Laboratory testing

HIV-DR genotyping was done at the Department of Medical Microbiology of the Academic Medical Center of the University of Amsterdam. All the other laboratory tests were conducted at the National Reference Laboratory in Kigali

#### Blood samples

For the chronically infected individuals recruited from the VCT site and eligible for ART, the analysis was done on baseline samples, i.e collected just before the initiation of ART. For the chronically infected women recruited from the cross sectional survey, the analysis was done on the sample collected at the time long-term infection was diagnosed. For the recently infected persons identified from the cross sectional survey, the analysis was done on the sample collected at the time recent infection was diagnosed. For the recently infected women selected from the longitudinal HIV incidence study, the laboratory investigations were done on a sample collected within six month after seroconversion

Five mL of whole blood was collected in EDTA vacutainer tubes (Becton Dickinson, Franklin Lakes, NJ). Fifty µL of fresh blood was used for CD4^+^ T cell enumeration. Subsequently and within four hours after sample collection, plasma was separated from the cellular fraction by centrifugation and collected into three aliquots of one mL. Plasma was stored at −80°C until further analysis. Transportation of frozen plasma to the Netherlands was done according to international standards of packaging, shipping and IATA recommendations.

#### CD4 count

CD4^+^T lymphocytes counts were measured in whole fresh blood on a single flow-cytometry platform using TruCOUNT® tubes on a FACScalibur instrument (Becton Dickinson, San Jose, CA, USA), according to manufacturer's instructions.

#### HIV-1 viral load

Plasma HIV-1 RNA (pVL) quantification was performed on thawed plasma using the Roche CobasAmpliPrep/Cobas TaqMan HIV-1 test (Roche Molecular Systems, France), according to the manufacturer's instructions. The lower limit of detection was 40 copies of HIV RNA/ml.

#### HIV-1 DR genotyping

HIV-1-DR genotyping was performed using the FDA-licenced ViroSeq genotyping kits (Abbot Molecular Inc, IL, USA) and an ABI automated sequencer (Applied Biosystems, Carlsbad, CA, USA), according to the manufacturer's recommendations. The quality of the *RT* and *protease* gene sequence data was assessed using the Sequence Quality Assessment of the Stanford University HIV Drug Resistance Database (http://sierra2.stanford.edu/).

The PR and RT sequences were screened for mutations associated with drug resistance using the ViroSeq v5.4 software. After editing, data were submitted to the Stanford University database and categorized according to the WHO list of mutations for surveillance of transmitted drug resistant HIV strains (2009 update) [32].

### Phylogenetic analysis

Sequences were aligned with the CLUSTAL W sequence alignment tool implemented in BioEdit Sequence Alignment Editor Version 7.0.9. The alignments were manually adjusted to preserve in-frame insertions and deletions. Phylogenetic analyses were performed with the MEGA5 software package distributed by Sudhir Kumar, Arizona State University, Tempe (MEGA 5 software package available at: www.megasoftware.net. (accessed February 8, 2011). Distances were estimated with the Kimura 2 method. Phylogenetic trees were generated with the neighbour-joining method and bootstrap resampling with 1000 replicates, using HIV-1 group M (A to K, CRF01-AE, and CRF02-AG) reference sequences from the Los Alamos database. Bootstrap values >80 were considered significant.

Sequences that could not be assigned to an HIV-1 subtype in the phylogenetic analysis were defined as unclassified (U) and further investigated for possible recombination events. Bootscanning analysis was performed in order to identify the presence of recombination breakpoints using SimPlot (version 3.5.1), a window size of 200 nucleotides, a step size of 20, and the Kimura (two-parameter) distance model. Putative parental strains of subtypes A and C were selected from our own data. The outgroup strains subtype B, D, F1 and G were selected from the Los Alamos HIV database. The same sequences were analysed with the REGA HIV-1 subtyping tool on the Stanford database to confirm the recombination events.

From the phylogenetic trees we examined transmission events (defined as bootstrap values = 100) within our cohort as well as phylogenetic links (defined as bootstrap values >80) of the Rwandan strains with similar HIV-1 subtypes and subtype recombinants circulating in the five neighbouring countries. For this purpose, reference sequences derived from the five countries surrounding Rwanda were selected from Los Alamos HIV sequence database (Los Alamos National Laboratory HIV Databases [http://hiv-web.lanl.gov]. All available *pol* fragments with a length of at least 1280 nucleotides, a known collection date and a classification into subtypes and inter subtype recombinant relevant for the Rwandan context, were retrieved from the database. Only one sequence per single individual was selected. For practical reasons, the analysis was done in three separate phylogenetic trees with sets of reference sequences of the same genotype(s).

### Statistical analysis

Baseline characteristics were reported as percentages for categorical data. The normality of data distribution was assessed by the Kolmogorov Smirnov test. Continuous variables were reported as mean with standard deviation or median with interquartile range (IQR) or range. The Pearson chi-square test was used to investigate whether the distribution of categorical data differed between groups. For numerical data, differences between groups were assessed using the student-t test or ANOVA test (for multiple groups) in case of normal distribution. For data not normally distributed, differences between groups were analyzed using the Mann-Whitney U test or the Kruskall Wallis for more than two groups.

## Results

### Study population

**Figure 1:**
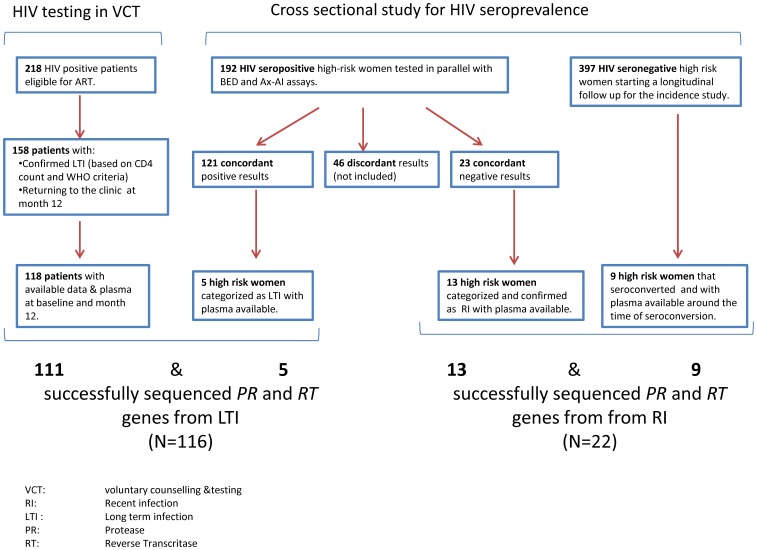
Selection of recently and chronically infected participants. Left: chronically-infected patient were selected from two sources: ·118 of 218 HIV patients eligible for ART and identified through VCT, were selected for HIVDR genotyping based on the completeness of the month 12 follow up visit, the viral load data and the availability of plasma. For seven samples, a *pol* sequence could not be generated during HIV-DR genotyping. ·from the cross sectional survey, five of 121 female participants classified as long-term infections had plasma available for HIVDR genotyping. In total, 116 HIV sequences of chronically infected participants were available for the analysis. Right: out of the patients participating in the cross sectional survey, 13 recent infections based on concordant negative results of the BED/Ax-AI assays were identified and had enough plasma available for HIVDR genotyping. Nine seroconverters were identified from the subsequent longitudinal incidence study and tested for HIVDR genotyping. In total, 22 *pol* sequences could be generated from recently infected individuals.

Two hundred and eighteen patients were identified with HIV infection and were immediately eligible for ART at the VCT site (figure 1). Of these, 158 completed their month 12 follow up visit and fulfilled the criteria for long term infection based on the presence of any of the following: CD4 count <350 cells/mL, WHO stage≥II or first positive HIV serology more than six months ago and as described in the method section. Paired VL data at baseline and month 12 as well as adequate volume of plasma for the laboratory investigations were available in 118 patients. One hundred and eleven out of 118 plasma subjected to HIV-DR genotyping yielded a PCR product and were included in the analysis.

Eight hundred high risk women participated in the cross-sectional survey at Projet Ubuzima. One hundred and ninety two women had a positive HIV serology and 190 were tested in parallel with the BED and Ax-AI assays (figure 1). One hundred and twenty one were classified as long-term infections based on simultaneous positivity with the two serological assays. Twenty three were classified as recent infections based on simultaneous negativity with the two assays. Forty-six samples with discordant results with the BED/Ax-AI algorithm were categorized as indeterminate and excluded from the analysis. Out of the 121 long term infections, five patients had adequate volume of plasma available for HIV-DR genotyping, had a PCR product generated and were all included in the analysis. Thirteen patients out of the 23 recent infections had adequate volume of plasma available for HIV-DR genotyping, which all yielded a PCR product.

Of the 800 high risk women participating in the cross-sectional survey, 397 were confirmed HIV negative and were eligible for the subsequent longitudinal HIV incidence study [27]. Nineteen of these participants seroconverted during the two years follow-up (figure 1). Nine seroconverters had an adequate volume of plasma available within a six-month period from seroconversion to conduct HIV-DR genotyping (figure 1) and a PCR product was obtained for all samples.

A total of 116 *pol* sequences from chronically infected subjects and 22 HIV-1 *pol* sequences from recently infected individuals were included in the analysis.

### Comparison between chronically and recently infected participants

The clinical and demographic characteristics of the 116 chronically and 22 recently infected subjects are displayed in table 1. The data indicate that among the chronically infected group, men and women had comparable characteristics except for weekly income, which was significantly higher for men (median US$ 21 versus 8, p = 0.006) and for marital status showing a higher proportion of married (median = 65.3 versus 33.8%) and a lower proportion of widow(er)s (median = 15.2 versus 38.5%) among men as compared to women (p = 0.009). Although chronically infected individuals (men and women) reported HIV positive partners (56.8%) more frequently than recently infected women (4.8%, p<0.001), only 63% of them had knowledge about the HIV status of their partner compared to 100% of the women in the high risk group. Overall, recently infected high-risk women differed from the chronically infected population with respect to most of the demographic parameters reported, reflecting the difference in social status and behaviors of this group as compared to the general population. Significantly higher CD4 count (median = 648 versus 221 cell/mL, p<0.001) substantiated the earlier stage of infection of recently infected women as compared to the rest of the study population. Lower plasma VL (median = Log_10_ 4.1 versus Log_10_ 4.8 HIV-1 RNA copies/mL, p = 0.008) could indicate that a majority of recent infections were included in the study posterior to the peak of viral replication, when the immune response against HIV begins to mount and prior to the re-increase of viral load during the later stage of the disease.

**Table 1:** Characteristics of the study population.

	Chronically infected				Recently infected	
	Men	Women	P_1_	Total	Women	P_2_
	(N = 51)	(N = 65)		(N = 116)	(N = 22)	
Demographics						
**Age in years Mean (sd)**	38.4(7.5)	36.7(8.6)	0.25	37.5(8.2)	24.9(4.4)	**<0.001**
**Marital status (%)**			**0.009**			**<0.001** ^*1^
Never married	7(15.2)	15(23.1)		22(19.8)	16(72.7)	
Married	30(63.3)	22(33.8)		52(46.9)	1(4.6)	
Divorced	2(4.3)	3(4.6)		5(4.5)	4(18.2)	
Widow(er)	7(15.2)	25(38.5)		32(28.8)	1(4.5)	
**Reported HIV positive partner n(%)**	22(55.0)	20(58.8)	0.82	42(56.8)^*2^	1(4.8)	**<0.001** ^*1^
**Weekly income median USD (IQR)**	21(8–33)	8(0.2–21)	**0.006**	12.5(4–29)	18(13.25)	**0.007** ^*3^
**Household size median (IQR)**	4(3–6)	5(4.7)	0.13	5(3–6)	2(1–3)	**<0.001** ^*3^
**Education level n(%)**			0.05	^*4^		**0.002^1^**
Never went to school	0	6(9.4)		6(5.3)	3(13.6)	
Some primary school	9(18.0)	10(15.6)		19(16.7)	12(54.6)	
Completed primary school	19(38.0)	12(18.8)		31(27.2)	4(18.2)	
Some secondary school	16 (32.0)	23(35.9)		39(34.2)	3(13.6)	
Completed secondary school	4(8.0)	11(17.2)		15(13.1)	0	
Post secondary school	2(4.0)	2(3.1)		4(3.5)	0	
**Age at sexual debut**	19.5(18–21)	18(16–22)	0.52	18.5(17–21)	17(15–18)	**0.009** ^*3^
**median years (range)**						
**Presence of HIV symptoms n(%)**	21(42.0)	29(46.0)	0.18	50(44.3)	9(40.9)	0.77
Laboratory data						
**Baseline CD4 count**	168(116–267)	223(110–301)	0.14	211(110–280)	648(493–822)	**<0.001** ^*3^
**Median cell/mL (IQR)**						
**Baseline viral load**	4.9(4.5–5.3)	4.8(4.2–5.2)	0.23	4.8(4.2–5.2)	4.1(3.6–4.6)	**0.008** ^*3^
**Median log_10_RNA copies/mL (IQR)**						

*^1^ Fisher's exact test ^*2^ n = 74 ^*3^Mann-Whitney test ^*4^ n = 114.

P_1_ value comparing men versus women among the chronically infected group.

P2 value comparing chronically versus recently infected participants.

One hundred and sixteen chronically (51 men and 65 women) and 22 recently (all women) infected persons participated in the study. Data are reported as total number and percentages (%); median and interquartile range (IQR) or mean and standard deviation (sd). P1 compares men versus women within the group of chronically infected participant. P2 compares chronically versus recently infected participants. ^*1^ Difference between groups was calculated using the Fisher exact test. ^*2^ Only 74 chronically infected participants (63.7%) were aware of the HIV status of their partner. ^*3^ Difference between groups was calculated using the Mann-Whitney test. ^*4^ Education level was reported in 114 chronically infected participants (98.2%) infected participants.

### HIV-1 subtype classification and distribution among the Rwandan samples

**Figure 2:**
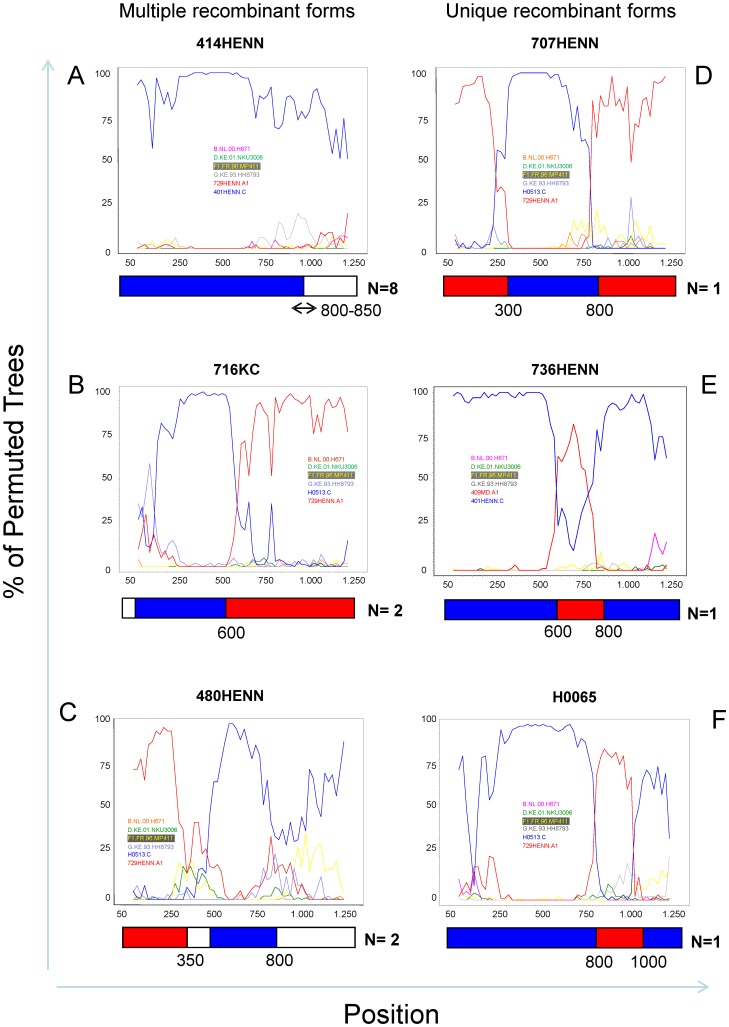
Bootscan analysis of RT and PR sequences of A/C recombinants from Rwanda. Bootscan analyses of representative multiple (left side: A, B, C) and unique (right side: D, E, F) A1/C recombinant forms. The analysis was performed using SimPlot 3.5.1 configured with 1000 bootstrap replicates, 200 basepair (bp) window and a step size of 20 bp. The x-axis shows aligned nucleotides of the sequences analyzed and the y-axis shows the percentage of permuted tree, i.e. the bootstrap value. The reference strains that were used in the analysis are indicated in color. Parental reference sequences, subtype B, D, G, and F1, were selected from the Los Alamos database and parental subtype A1 (729HENN) and C (H0513) sequences were taken from this study. The breakpoint position(s) and the subtype designation is shown schematically at the bottom of each bootscan, with the number of sequences harboring a similar recombination pattern indicated in bold (e.g, figure A, N = 8). 414HENN is an example of a group of 8 similar recombinants (A). 716KC & 427HENN (B) and 480HENN & 754HENN (C) shared similar bootscan patterns. Graphs C to F show results for observed unique recombinant variants.

In total, 111 out of 138 sequences could be assigned a pure HIV-1 subtype. Twenty seven variants could not be classified initially and were further analyzed by bootscan analyses. Out of these, 25 sequences were characterized as A1/C recombinants. Thirteen of these showed a unique recombination pattern between subtypes A1 and C, whereas similar recombination pattern were found in one group of eight and two groups of two individuals, respectively (see figure 2 A, B, C). One of the last two sequences without an assigned HIV-1 subtype was characterized as A1/D (HENN730) recombinant and the other remained unclassified (H0172, data not shown). These results were confirmed using the REGA HIV-1 subtyping tool on the Stanford database.

**Figure 3:**
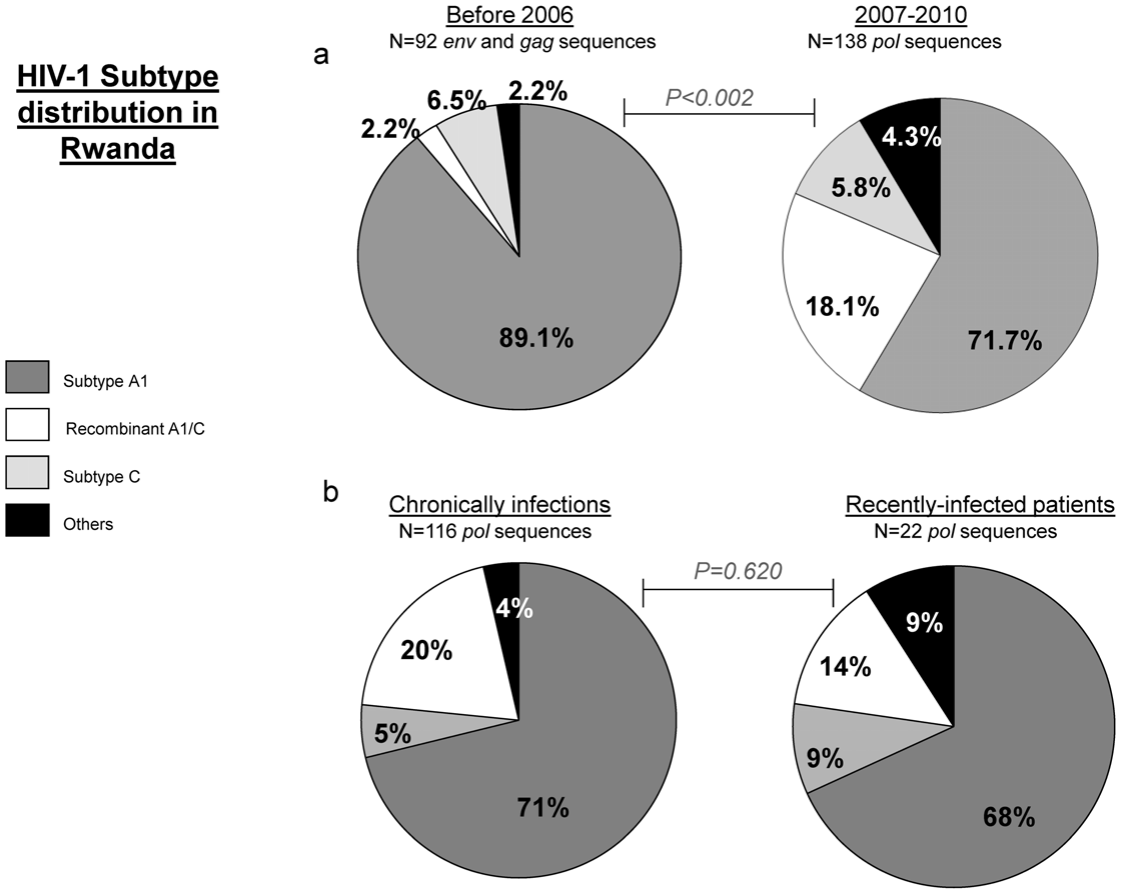
HIV-1 subtype distribution in Rwanda. a) The pie chart on the left shows the HIV-1 subtype distribution of 92 *gag* and *env* sequences isolated between 1988 and 2006 in Rwanda and available from the Los Alamos database. The classification of subtype A into the A1 sub-subtype was not routinely done before 2006. The pie chart on the right shows the HIV-1 subtype distribution of 138 *pol* sequences from the chronically and recently infected study participants enrolled between 2007 and 2010. The HIV-1 subtype distribution before 2006 and in our cohort was significantly different as calculated by the Pearson Chi square (p<0.002). b) Subtype distribution in chronically infected study participants (on the left) and recently (on the right) infected participants enrolled in our study between 2007 and 2010. No statistically significant differences in the subtype distribution were found.

The HIV-1 subtype distribution among the Rwandan samples indicated a majority of A1 (71.7%), followed by A1/C recombinants (18.1%), C (5.8%), D (2.9%), one A/D recombinant (0.7%) and one ‘U’ subtype (0.7%, figure 3a).

HIV-1 subtypes were similarly distributed among recently as compared to chronically infected persons (figure 3b). No chronically-infected woman from the normal risk group were identified within the population infected by subtype D, A/D recombinant and ‘U’. The age distribution among each subtype indicated a lower median age among subtype D-infected (24 years) as compared to subtype A1-infected (37 years) persons, but this difference was not significant (p = 0.053, data not shown).

In order to determine whether changes in subtype distribution occurred over time, we analyzed the subtype distribution of HIV sequences available from the Los Alamos database and isolated before the initiation of the present study. A total of 92 unique sequences (80% *env* and 20% *gag* sequences) collected in Rwanda between 1988 and 2006 were selected from the Los Alamos database. The subtype distribution showed a large predominance of subtype A and A1 (89.1%) and modest frequencies of subtype C (6.5%), A/C recombinants (2.2%) and others subtypes (2.2%, figure 3a). Proportions of HIV-1 subtypes within HIV sequences isolated before 2006 were significantly different from the distribution observed in our cohort (Pearson Chi-square p<0.002), mainly showing a spread of A1/C (18.1% in 2010 versus 2.2% before 2006) recombinant strains over the parental subtype A (71.7% in 2010 versus 89.1% before 2006), while the proportion of C strains remained relatively stable (5.8% in 2010 and 6.5% before 2006).

### Frequency of transmitted HIV drug resistance mutations in the Rwandan cohort

Of the 138 sequences analyzed, four (2.9%) showed evidence of transmitted HIV drug resistance mutations (data not shown). Two sequences presented NNRTI mutations only and two sequences had simultaneous NNRTI and NRTI mutations. Two variants with HIV drug resistance mutation were classified as subtype A1 and two as subtype C and three out of the four sequences were derived from women with no self-reported history of PMTCT. All four sequences with HIV drug resistance mutations were derived from the chronically infected patients.

### Relationships between viruses infecting the Rwandan cohort

**Figure 4:**
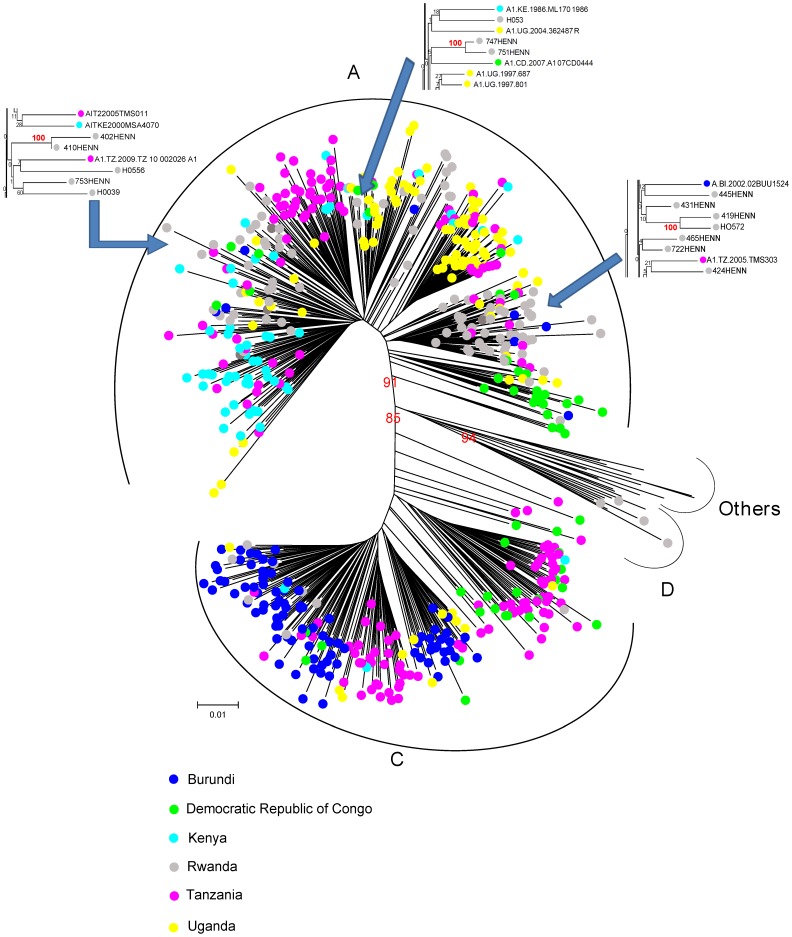
Phylogenetic tree for subtype A1 and C. HIV-1 A1 (N = 94) and C (N = 8) *pol* sequences obtained from the Rwandan study participants were analyzed in connection to reference sequences from the Los Alamos database originating from Rwanda (N = 4) and the five surrounding countries collected between 1992 and 2009 (272 A1 and 212 C). The four subtype D (analysis 2, data not shown) and the 25 A1/C recombinant sequences were further examined in two separate analyses including 376 subtype D reference sequences and 6 A1/C reference sequences, respectively (analysis 3 data not shown). Country of origin of each sequence is color coded, with Rwanda in grey. The bootstrap values >85% are indicated in red. Related sequences found within subtype A1 (bootstrap value 100) are shown in detail in the three rectangular phylogenetic tree zoomed inserts.

The relationship between the Rwandan HIV-1 sequences was investigated. We identified three pairs of individuals carrying related subtype A1 viruses. i.e with bootstrap value of 100 (figure 4, zoomed inserts): **1)** two chronically infected women, **2)** two chronically infected men, **3)** one chronically and one recently infected woman. The possibility of direct virus transmission between individuals carrying related viruses was not supported neither by phylogenetic data nor by the same gender character of the patient pairs.

### Routes of HIV-1 transmission between Rwanda and neighboring countries

The possibility of exchange of HIV-1 variants between the neighboring countries and Kigali was examined by comparing the Rwandan *pol* sequences with sequences from the five neighboring countries selected from the Los Alamos database. Eight hundred and sixty-six *pol* sequences collected between 1992 and 2009 were included in the analysis. The number, origin and sampling year of the reference sequences are depicted in table 2. The analysis was done separately for **1)** subtype A or A1& C; **2)** subtype D and **3)** A1/C recombinants.

**Table 2:** Reference *pol* sequences from the Los Alamos database included in the phylogenetic analyses.

	HIV subtypes							
	A or A1		C		D		A1/C	
Countries	N	Sampling year(s)	N	Sampling year(s)	N	Sampling year (s)	N	Sampling year (s)
Burundi	8	2002	92	2002	2	2002	0	
Democratic	29	1997,2002	23	2002, 2007	20	1983–1985,	1	2002
Republic		2007				1997, 1998		
of Congo						2002, 2007		
Kenya	55	1986, 1994	3	1991, 2000,	11	1993, 1996	0	
		1997, 1999–2002		2004		1999, 2001, 2006		
		2004–2007						
Rwanda	4	1992, 1993	0		0		2	1992
Tanzania	83	1997, 2001, 2005	82	1997, 1998,	64	2001, 2005,	2	2005
		2007–2009		2001, 2002,		2007–2009		
				2005, 2007–2009				
Uganda	93	1985, 1992,	12	1997, 2002,	276	1991, 1994,	1	2003
		1997–1999,		2004		1997–2005		
		2004–2006						
**Total**	**272**		**212**		**376**		**6**	
	Analysis 1 (figure 4)				Analysis 2		Analysis 3	
					(data not shown)		(data not shown)	

1480 n long references sequences of the subtype identified in the Rwandan cohorts were selected from the Los Alamos database. The table indicates the HIV-1 subtypes, respective number of sequences, country of origin and year of sampling. In earlier years subtype A was not yet subdivided into sub-subtype A1. The phylogenetic analysis was done in three separate trees involving subtype A or A1 and C (analysis 1 also shown in figure 4), subtype D (analysis 2) and A1/C recombinant (analysis 3).

In the first analysis no country-specific clusters were found for subtypes A1 and C variants. The Rwandan sequences were distributed throughout the reference sequences from the neighboring countries with no evidence for direct relationships (figure 4).

The phylogenetic analysis of the four Rwandan subtype D and the 25 A1/C recombinant variants identified in our cohort did not show evidence of clustering with the reference sequences from the Los Alamos database (data not shown).

Thus, Rwandan strains sequences could not be linked to any of the strains isolated from the surrounding countries included in the analysis. No time-associated trend could be identified.

## Discussion

Our aims were to gain insight into the situation and the trends of the current HIV-1 epidemic in Rwanda. We set out to describe the genetic subtype distribution of HIV-1 strains infecting ART-naïve patients from Kigali and estimate its evolution over time in connection with the HIV-1 epidemics of the five surrounding countries.

Our data suggest that while subtype A1 remains the predominant HIV clade circulating in Kigali, the distribution of HIV-1 subtypes has evolved from the situation previous to 2006. Notably, we found a higher percentage of *pol* A1/C recombinant viruses in our cohorts, as compared to the frequencies observed in *env* and *gag* sequences isolated in Rwanda before 2006 and available from the Los Alamos data base. Very few *pol* sequences derived from sub-Saharan African patients have been generated during the pre-ART era. Although subtype classification might be discordant in different parts of the genome, comparing *gag* and *env* to *pol* sequences provides an approximation of changes in HIV-1 subtypes distribution over time. The indication of a possible shift in HIV-1 subtype distribution in Kigali was not corroborated by any significant differences in HIV-1 subtype distribution between the groups of recently versus chronically infected persons. The false recent rate associated with the combined BED/Ax-AI algorithm in the present study population is reportedly low (2.1%) [27], with no expected ambiguity in the characterization of recent and long term infections in our study. However, the actual times of infection for each patient might still be too adjacent, with the lack of clear-cut differences in the subtypes of viruses infecting the two groups. Alternatively, the changes in HIV-1 subtype distribution over time could be related to some biases in the selection of HIV sequences in our study and those available in the Los Alamos database, which were not primarily chosen to be representative of the HIV-1 distribution in Rwanda at a particular time point. Interestingly, the trends towards an expansion of A1/C recombinants confirmed the results of Servais et al on a group of 43 HIV-1 infected pregnant women recruited in Kigali in 2000 [21].

The four subtype D strains identified in our cohort were shown to be associated with relatively younger individuals, as compared to other subtypes. Two of these patients were male and two were females from the high risk behavior groups, with no clear self-reported socio-demographic links. Although younger age represents a correlates of new HIV infections [33], the possible implication of subtype D in relatively more recent infections remains speculative, given the limited number of subtype D infections identified in this study.

The further characterization of recombination events in our cohort suggested at least two mechanisms underlying the increased frequency of A1/C recombinants. On one hand, the identification of three similar patterns of recombination in 12 patients suggests that A1/C variants spread through *de novo* infections. On the other hand the characterization of 13 unique recombination patterns indicates that random recombination events within the *pol* gene are ongoing upon co-infections of individual patients with parental A1 and C strains. The possibility of an accidental emergence of A1/C recombinants from circulating A1 and C parental strains seems unlikely given the relatively low frequency of C strains in Rwanda from 1992 (6.5%) throughout 2010 (5.8%). Moreover, the apparent spread of A1/C recombinants in our cohort contrasts with the epidemiological evidence for the dominant spread of subtype C strains over the other HIV variants in Africa, India, China and South America [34]. A growing body of evidence suggests that the asymmetric spread of certain HIV subtypes in some regions of the world is associated with a superior replicative fitness and higher efficiency of transmissibility [35]–[39]. A1/C recombinant strains are reported to be emerging in South Africa where subtype A1 is rare [40]. Interestingly a unique A1/C recombinant was also described in Canada, closely related to another A1/C variant from Rwandan origin [41]. Altogether, these observations suggest that A1/C recombinant strains may display biological advantages over their parental A1 and C strains. Further research is needed to assess the epidemiological significance of A1/C recombinants.

Although the study participants were all coming from Kigali and its surroundings the analysis did not support neither a common origin nor the possibility of any direct transmission of the HIV subtypes and subtype recombinants identified in the cohorts. Three pairs of participants with potential epidemiological links were identified but no large networks of transmission could be documented within the present data set. Clusters of HIV transmission reflect a “founder effect” which is commonly observed within specific risk groups such as intravenous drug users (IDUs) or men who have sex with men (MSM) [42]–[45]. In populations where HIV transmission occurs mainly through heterosexual contacts_like in Rwanda, evidence for transmission clusters is generally more limited [46]. This could be related to the existence of complex sexual networks, but also to sampling issue. Study populations recruited in areas of relatively high HIV prevalence are often sparsely sampled (albeit being representative) with a reduced probability of including patients belonging to the same transmission network(s) and a bias toward the underreporting of infection clusters [42]

The lack of close relationship between the HIV sequences from patients in our cohort highlights the complexity of HIV dissemination networks, which represents a challenge for HIV prevention. In addition, our data underscores further social barriers to HIV control. First, only half of the study participants knew the HIV status of their partner(s). Second, there was no partnership history amongst the study participants. This suggests that HIV status remained frequently undisclosed within relationships and that partners do not tend to seek treatment in the same health care facility. Our observations are in accordance with previous reports from Rwanda and other sub Saharan countries, emphasizing the fear of partner reaction and an overall stigma associated with HIV infection in the community [47]. In a context where the majority of HIV transmission occur between co-habiting partners [48], this observation has important implications for HIV prevention and care.

The ART programme was initiated in Rwanda in 2004. The level of baseline HIVDR mutations under 5% is in the range of what has been described in countries where ARVs are available for a comparable duration of time [49]–[51]. This low frequency of transmitted HIV is an indirect indicator of the correct use of ART in Rwanda [52], confirming the virologic efficacy of the first line recommended regimen [24]. The data do not provide evidence of exchange of HIV strains harboring drug resistance mutations with Uganda, a current hot spot of emerging HIV-DR [23].

Given the frequent population movements traditionally taking place in the Eastern African region [6]–[8], we postulated that migration might (have) play(ed) a role in the past and present HIV epidemic in Rwanda. We did not find evidence that any of the HIV variants identified in our cohort clustered with the corresponding subtypes or subtype recombinants from the five near-by countries. The possibility that subtype D strains have been recently introduced from Uganda _where it largely circulates [53], [54] to Kigali was not substantiated by bootstrap analyses, involving no less than 276 Ugandan reference D strains. With Kigali being the largest city of the country and with the highest HIV-1 prevalence [5] our study population might illustrate the situation of HIV diversity at the national level, although inference of the results to the situation at the national level needs to be drawn with caution given the limited size of our sample. Notwithstanding, our observations suggests the notion of a fenced, independent evolution of the Rwandan epidemic with little influence of cross border migration events. However, given the uneven availability of up-to-date *pol* sequences from East and Central Africa in the Los Alamos database, our results may have been biased. Therefore, the existence of genetic links between HIV strains from Rwanda and surrounding countries cannot totally be ruled out.

In conclusion, the data presented here indicate that in Kigali, the HIV-1 epidemic is mainly driven by A1 variants, evolves toward a clear spread of A1/C recombinants and a possible minor emergence of subtype D strains among younger individuals. The present set of data did not show evidence of a major influence of population migrations in and out the five main surrounding countries on the current HIV epidemic in Rwanda. Future phylodynamic analyses of high-density population samples may be envisaged as contemporary HIV-1 sequences from Rwanda and surrounding countries become increasingly available. These studies will contribute to a more highly-defined description of HIV-1 transmission networks in Rwanda.

Our report highlights the relatively low level of primary HIV-DR mutations in the country suggesting the good performance of the ART program in Rwanda. However, the data also indicate that national HIV prevention strategies may benefit from more focus on health education and behavioral interventions that include promotion of couples' counseling, testing and disclosure.
